# Near‐Infrared Spectroscopy Sensors Placed Frontally and Parietally to Detect Large Vessel Occlusion Stroke

**DOI:** 10.1155/nri/7575719

**Published:** 2026-11-09

**Authors:** Joonas Kiili, Pauli Vuorinen, Jyrki Ollikainen, Pasi Jolma, Heini Huhtala, Piritta Setälä, Sanna Hoppu

**Affiliations:** ^1^ Faculty of Medicine and Health Technology Tampere University Tampere Finland uta.fi; ^2^ Emergency Medical Services Centre for Prehospital Emergency Care Wellbeing Services County of Pirkanmaa Tampere Finland; ^3^ Department of Anaesthesiology Hospital Nova Wellbeing Services County of Central Finland Jyväskylä Finland; ^4^ Department of Neurosciences and Rehabilitation Tampere University Hospital Tampere Finland pshp.fi; ^5^ Faculty of Social Sciences Tampere University Tampere Finland uta.fi

**Keywords:** large vessel occlusion, near-infrared spectroscopy, NIRS, stroke, thrombectomy

## Abstract

**Background:**

We aimed to determine whether two‐channelled near‐infrared spectroscopy (NIRS) was feasible for recognising anterior large vessel occlusion (LVO) with a device suitable for prehospital use and whether probe placement (frontal vs. parietal) made a difference.

**Methods:**

We analysed NIRS readings of six computed tomography angiography (CTA)‐confirmed LVO‐stroke patients at Tampere University Hospital during the six‐month study period. We placed four sensors on patients’ heads: two frontally and two parietally. We provided a detailed description and analysis of these recordings and their correlation with imaging‐confirmed changes in perfusion.

**Results:**

In the frontally placed sensors, the rSO_2_ was 1–8 percentage points higher on the affected side before and 0–9 percentage points higher on the affected side after endovascular treatment (EVT). rSO2 values did not change significantly. One patient showed higher rSO_2_ on the healthy side (5 percentage points) before and after EVT. We could not recover frontal values from one patient. In parietally placed censors, all patients had 3–9 percentage points higher rSO_2_ on the healthy side before EVT. After EVT, 3 patients had 3–7 percentage points higher rSO_2_ on the healthy side. In two cases, after EVT, the difference in rSO_2_ changed to 1‐2 percentage points higher on the affected side. We could not record parietal readings in one patient.

**Conclusions:**

Neither NIRS sensor placement detected differences in rSO_2_ in LVO; therefore, NIRS appears unsuitable as a diagnostic tool for LVO. Future research should focus on other technologies.

## 1. Introduction

Worldwide, stroke is the second leading cause of death and the third leading cause of permanent disability [1, 2]. About 12%–35% of all strokes are large vessel occlusions (LVOs). LVO is associated with a more dismal prognosis among strokes. Approximately 10%–68% of LVO patients would benefit from endovascular treatment (EVT) [3]. The most crucial modifiable prognosis factor in LVO is the time between symptom onset and treatment [4].

Incorrect patient referral to a hospital incapable of EVT adds delay [5]. A good diagnostic test for LVO detection must have high sensitivity so that no patients with LVO are missed and high specificity to prevent overloading emergency medical services and EVT‐capable centres. The test should also be fast and easy to apply to be usable in a prehospital environment [6]. Different clinical scoring systems for LVO detection have been developed to aid patient referral. Sensitivity and specificity vary widely across these tests. Currently, clinical scores alone are not enough to reliably detect LVO strokes [6–8]. Various technologies have been suggested and researched to aid the detection of LVO, such as telemedicine, mobile stroke units, blood samples, ultrasound, microwaves, volumetric impedance phase shift spectroscopy, transcranial Doppler, near‐infrared spectroscopy (NIRS) and electroencephalography [6, 9, 10].

NIRS is a non‐invasive method to evaluate regional oxygenation and perfusion initially developed in the ‘70s [11]. An NIRS device has a sensor composed of a diode or laser light source emitting light with a wavelength between 700 and 1000 nm and a detector receiving the light not absorbed by tissues. Major absorbers of near‐infrared light are haemoglobin, myoglobin, bilirubin and cytochrome oxidase. NIRS calculates the regional oxygen saturation (rSO_2_) from the absorbed light [12]. Notably, the saturation value combines cerebral arterial, capillary and venous blood saturations. Approximately 75%–80% of the value originates from venous blood [11, 12]. NIRS has previously been used to monitor cerebral saturations and perfusion during carotid endarterectomy, thrombolysis, EVT and the sub‐acute phase of stroke in the ward [13–20]. NIRS has been successfully used in the prehospital setting to study changes in cerebral oxygen saturations during prehospital anaesthesia. In the BOPRA (Brain Oxygenation During Prehospital Anaesthesia) study, they used one probe placed on the left side of the forehead [21].

A few small studies on using NIRS to detect LVO and monitor during EVT have shown preliminary results that NIRS could help detect LVO and monitor during EVT [13–15, 22]. Hiramatsu et al. showed that frontally placed probes showed a significant difference in patients with internal carotid artery (ICA) occlusion but failed to show a difference in patients with distal middle cerebral artery (MCA) occlusion. They hypothesised that parietally placed probes could show the interhemispheric difference better than frontally placed probes, but this might negatively impact imaging during EVT [15]. Our goal was to find a device suitable for prehospital use that reliably detects LVO in stroke patients. In this pilot study, we aimed to investigate whether continuous‐wave NIRS is a feasible instrument for recognising anterior LVO and its recanalisation. We also hypothesised that parietal probe placement would be superior to the conventional frontal position, as the sensor would be more precisely over the ischaemic brain tissue.

## 2. Methods

### 2.1. Study Design

This was a non‐randomised prospective case series study of emergency department (ED) patients with computed tomographic angiography (CTA)‐confirmed LVO stroke. This was a feasibility study to determine if a larger study on a prehospital patient population was warranted. We aimed to recruit 10 patients.

### 2.2. Setting

The ED at Tampere University Hospital serves as the only 24/7 specialised care centre for 530,000 people. It is also a comprehensive stroke centre for neighbouring hospital districts with a population of 1,000,000 inhabitants. Our institution performs almost 200 EVTs yearly.

### 2.3. Study Population

Patients in the ED with computed tomography‐confirmed LVO were included if treated by an on‐call neurologist with training and experience in NIRS. Patients under 18 were excluded. We aimed to collect NIRS data from 10 patients with CTA‐confirmed LVO to identify a positive signal that would justify a larger study in the prehospital setting. We stopped recruitment after six patients because the importer couldn’t provide enough sensors.

### 2.4. Data Collection

We used an NIRS device (Nonin SenSmart X‐100M), suited for prehospital use, to collect data on regional brain tissue saturation changes and interhemispheric differences during stroke and immediately after treatment. We used the SenSmart model 8204CA sensor, which has source‐detector separation distances of 20 and 40 mm and can detect oxygen saturation at a depth of approximately 20 mm. No signal preprocessing, artefact rejection or noise filtering methods were applied during or after data collection. Data were collected between July 2022 and January 2023. For all study patients, four NIRS sensors were placed on the head (bilaterally on the forehead and parietally). Frontal sensors were placed over each eyebrow, and parietal sensors were placed on an imaginary line between the patient’s ear canals, approximately at the midpoint on each side (Figure 1). Sensor placement was intentionally kept as simple as possible, with future research in time‐critical patients in a prehospital setting in mind. Hair was removed from the area of the parietal sensors. Data recording started in the CT scan room after the LVO was confirmed and continued through the EVT to the stroke unit. The NIRS device recorded rSO_2_ values every 4 s. Unfortunately, we had missing data from patient #3’s right forehead and patient #6’s parietal sensors, most probably due to poor adherence. An experienced interventional radiologist assigned patients an expanded treatment in cerebral infarction (eTICI) score based on digital subtraction angiography images obtained immediately after EVT. A control non‐contrast head CT was taken within 24 h of admission from every stroke patient receiving EVT. None of the study patients had intracranial haemorrhage.

**FIGURE 1:**
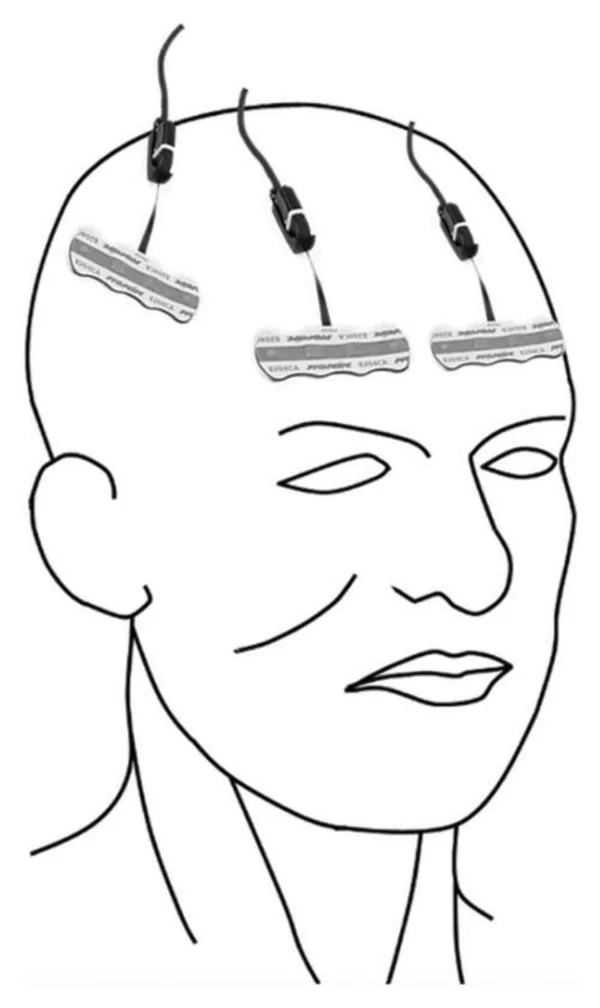
Positioning of the sensors on the patients’ head.

### 2.5. Analysis

We analysed the data from all sensors 5 min before and after the EVT. Statistical testing was deemed unnecessary due to the small number of patients and the high interindividual variation in average rSO2 values. We decided to provide a detailed description and analysis of the six NIRS recordings to explain how these findings correlated with the imaging‐confirmed changes in perfusion. All data are presented as medians and interquartile ranges (IQR) or numbers (%).

### 2.6. Role of the Funding Source

The funder of the study had no role in study design, data collection, data analysis, data interpretation or writing of the report.

## 3. Results

We recruited six patients in total. Four patients (67%) were male. The median age was 82 years (range: 54–88 years). The most common chronic diseases among the patients were hypertension (50%) and hypothyroidism (50%). Three patients had occlusion of the right MCA, one of the right ICA, one of the left MCA and one had multiple occlusions. Three patients received EVT within 2 h of the last known well (LKW). For two of the patients, we could not determine LKW. EVT results ranged from eTICI score 2a to 3. The patients’ recovery varied significantly. The modified Rankin Scale (mRS) 90 days after the EVT ranged from 0 to 4. One patient was lost to follow‐up. Table 1 summarises patient characteristics, and Table 2 shows changes in interhemispheric differences in rSO_2_. All patients were treated under remifentanil sedation and had spontaneous breathing. All had oxygen saturation of at least 95% and maintained a mean arterial pressure (MAP) over 90 mmHg, either spontaneously or with norepinephrine infusion.

**TABLE 1:** Patient characteristics.

ID	Age (years)	Gender	Chronic diseases	Site of occlusion	Presenting symptoms	Time from LKW to recanalisation	mRS after 3 months
1	83	Male	Hypertension	Right ICA	Hemiparesis, dysarthria and CED	10 h	3
2	80	Male	Hypertension, hypothyroidism	Right MCA (M1‐M2)	Hemiparesis and CED	2 h	2
3	88	Female	Hypothyroidism, cataract	Right MCA (M2)	Hemiparesis, facial asymmetry, global aphasia and CED	Unknown	Unknown
4	87	Male	Hypertension, COPD, obstructive sleep apnoea, atrial fibrillation	Right MCA (M1)	Hemiparesis and CED	2 h	1
5	78	Female	Hypothyroidism	Left MCA (M1)	Hemiparesis, global aphasia and CED	2 h	0
6	54	Male	Psoriasis, cervical spinal stenosis	Right ICA, ACA and PCA	Hemiparesis, global aphasia and CED	Unknown	4

**TABLE 2:** Changes in rSO2 interhemispherical difference.

ID	Site of occlusion	Frontal rSO_2_ interhemispherical (affected side)			Parietal rSO_2_ interhemispherical (affected side)		
		Before	After	Change	Before	After	Change
1	Right ICA	Higher	Higher	No change	Lower	Lower	No change
2	Right MCA (M1‐M2)	Higher	No difference	rSO_2_ equalised	Lower	Lower	No change
3	Right MCA (M2)	N/A	N/A	—	Lower	Higher	rSO_2_ improved
4	Right MCA (M1)	Higher	Higher	No change	Lower	Lower	No change
5	Left MCA (M1)	Lower	Lower	No change	Lower	Higher	rSO_2_ improved
6	Right ICA, ACA and PCA	Higher	Higher	No change	N/A	N/A	—

Patient 1 was an 84‐year‐old male with arterial hypertension as a pre‐existing condition. He was previously independent in activities of daily living, but he spent the last four days admitted due to pneumonia. Presenting symptoms included hemiparesis, dysarthria and conjugate eye deviation (CED). The CTA showed an intracranial occlusion of the distal part of the right ICA and considerable penumbra. EVT was performed rapidly with extensive recanalisation (eTICI 2c) approximately 10 h after the LKW. The patient recovered; three months after the intervention, he lived independently (mRS 3).

In the frontal sensors, rSO_2_ was a median of 8% points (IQR 8‐9) higher on the affected side before the EVT and 9% points (IQR 8‐9) higher after the EVT compared to the unaffected side. In the parietal sensors, the median rSO_2_ was 3% points (IQR 1–4) higher on the healthy side before and 3% points (IQR 2.5–4) after the EVT compared to the affected side. Figure 2A,B shows the changes in saturations between forehead and parietal sensors, respectively.

**FIGURE 2:**
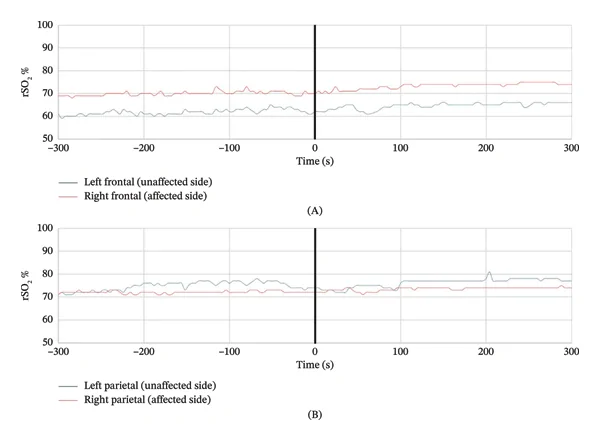
(A) Saturation changes in frontal sensors, Patient 1. (B) Saturation changes in parietal sensors, Patient 1.

Patient 2 was an 80‐year‐old male whose pre‐existing conditions included arterial hypertension and hypothyroidism. He had previously been treated for spontaneous venous thrombi but was not on active anticoagulation. Presenting symptoms were hemiparesis and CED. A tandem occlusion of the right MCA, distal M1 and proximal M2, was seen on CTA. The patient underwent immediate EVT, and complete recanalisation (eTICI 3) was achieved 2 h after LKW. The patient recovered to mRS 2 within three months.

The second patient’s forehead sensors showed rSO_2_ 3% points (IQR 2–5.5) higher on the affected side before EVT than on the unaffected side. After EVT, the difference was 0 (IQR ‐1‐1). Parietal sensors showed a median of 9% points (IQR 8, 5–9) higher rSO2 on the healthy side before EVT and 7% points (IQR 7‐8) higher after compared to the affected side. The changes in saturations between the forehead and parietal sensors are shown in Figure 3A,B, respectively.

**FIGURE 3:**
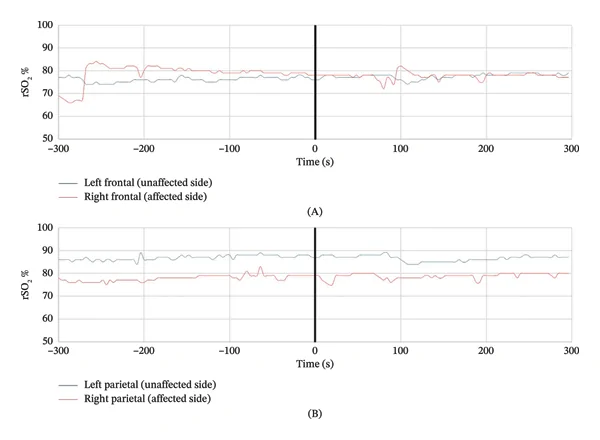
(A) Saturation changes in frontal sensors, Patient 2. (B) Saturation changes in parietal sensors, Patient 2.

Third patient was an 88‐year‐old female who had cataract and hypothyroidism as pre‐existing conditions. She was found by relatives with hemiparesis, facial asymmetry, global aphasia and CED after an unknown delay. Primary CTA showed a proximal occlusion of the MCA‐M2. Recanalisation was successful after three EVT attempts, and reperfusion of at least 50% of the ischeamic territory (eTICI 2b) was seen, but distal emboli were seen after the intervention. She was lost to follow‐up.

The right frontal sensor did not record any measurements at any point, so comparing frontal sensors was impossible. In the parietal sensors, rSO_2_ was a median of 4% points (IQR 4‐5) higher on the healthy side before the intervention compared to the affected side. After EVT, rSO_2_ was a median of 2% points (IQR 1–4) higher on the affected side than on the unaffected side. Figure 4 shows the changes in rSO_2_ in parietal sensors.

**FIGURE 4:**
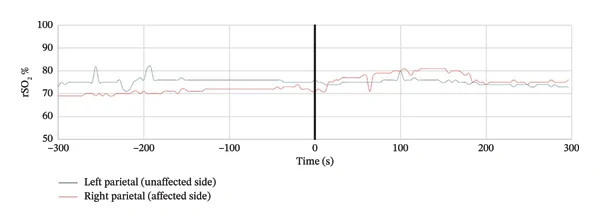
Saturation changes in parietal sensors, Patient 3.

Patient 4 was an 87‐year‐old male with a history of hypertension, chronic obstructive pulmonary disease, obstructive sleep apnoea and atrial fibrillation. Anticoagulation was waived 10 years earlier due to a subdural hematoma. The patient’s spouse called the ambulance after recognising stroke symptoms: hemiparesis, global aphasia and CED. A distal occlusion of the right MCA‐M1 was seen on CTA with no irreversible ischaemic damage. EVT was successfully performed 2 h after symptom onset, with partial reperfusion of the affected area (eTICI 2b). Some residual occlusion was seen in the second part of the right MCA. The patient recovered well and lived at home (mRS 1) three months after the intervention.

Frontal sensors showed a median rSO_2_ difference of 4% points (IQR 3‐4) higher on the affected side before and 6% points (IQR 5–11) higher after the intervention compared to the unaffected side. The median rSO_2_ in parietal sensors was 5% points (IQR 4–6) higher on the healthy side before the intervention and 4% points (IQR‐4‐5) higher after EVT compared to the affected side. Figure 5A,B shows the changes in rSO_2_ for the frontal and parietal sensors, respectively.

**FIGURE 5:**
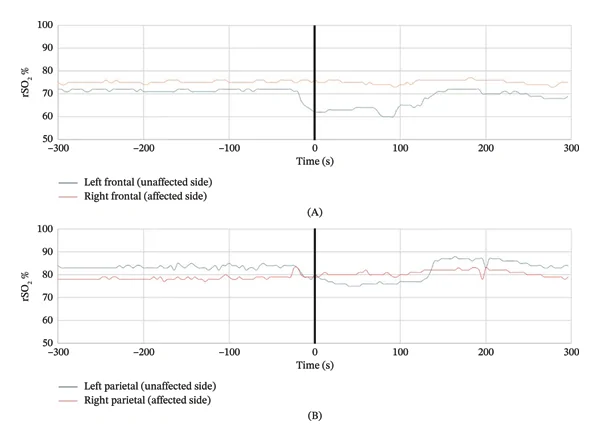
(A) Saturation changes in frontal sensors, Patient 4. (B) Saturation changes in parietal sensors, Patient 4.

Fifth patient was a 78‐year‐old female who had been previously treated for hypothyroidism. An emergency call was made after she had been seen falling. The paramedics found her speechless with hemiparesis and CED. CTA showed an occlusion in the MCA‐M1. During the EVT, a dissection was suspected and treated with an intra‐arterial stent 2 h after LKW. At least 50% reperfusion of the ischaemic area was detected after EVT (eTICI 2b). Three months later, she fully recovered (mRS 0).

Frontal sensors showed a median rSO_2_ 5% points (IQR 4‐5) higher on the healthy side before EVT and 5% points (IQR 3–6) higher after the intervention than on the affected side. In the parietal sensors, the rSO_2_ was a median of 5% points (IQR 3–7) higher on the healthy side before EVT and 1% point (IQR‐2‐3) higher on the affected side after the EVT compared to the unaffected side. Changes in the frontal and parietal sensors’ rSO_2_ are shown in Figure 6A,B, respectively.

**FIGURE 6:**
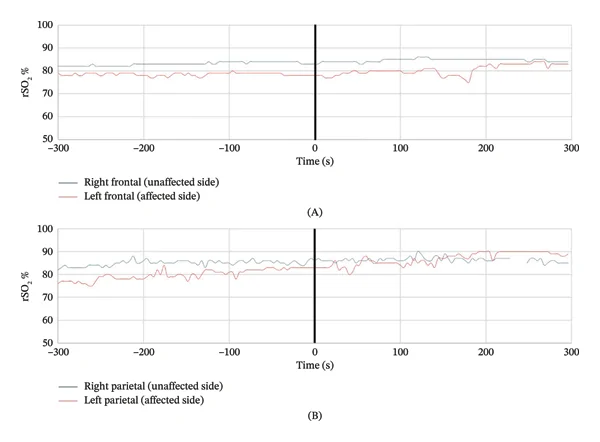
(A) Saturation changes in frontal sensors, Patient 5. (B) Saturation changes in parietal sensors, Patient 5.

Patient 6 was a 54‐year‐old male with psoriasis and cervical spinal stenosis. The patient woke up with a numb lower limb. After midday, he was found fallen on the floor. The paramedics noticed facial asymmetry, hemiparesis, speech difficulties and CED. CTA showed an occlusion in the right ICA. There were also occlusions in the pericallosal and posterior cerebral arteries. However, considerable penumbra was seen, and EVT was considered the treatment of choice. The carotid artery was recanalised, but distal emboli remained in M2. Anterior and posterior cerebral arteries remained occluded. Less than half of the affected area regained perfusion (eTICI 2a). After three months, the patient was still in a hospital ward (mRS 4).

Frontal sensors showed a rSO_2_ 1% point (IQR ‐1‐3) higher on the affected side before intervention and 5% points (IQR 2–9) higher on the affected side after intervention compared with the unaffected side. Due to poor sensor adhesion, we could not obtain readings from the parietal sensors or calculate the difference between them, neither before nor after EVT. Changes in frontal rSO_2_ sensors are shown in Figure 7.

**FIGURE 7:**
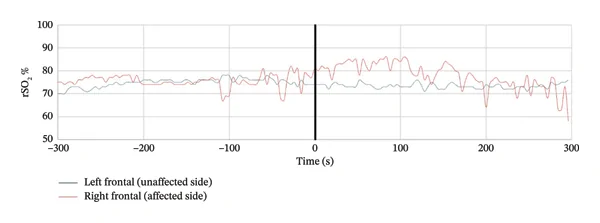
Saturation changes in frontal sensors, Patient 6.

## 4. Discussion

We did not find any clinically significant changes in rSO_2_ on the affected side before and after EVT, despite the eTICI score showing major or complete reperfusion in all but one patient. Parietally placed sensors were not superior in detecting interhemispheric differences in rSO_2_, although digital subtraction angiography images showed that the parietal sensors were located precisely over the area suffering from hypoperfusion during MCA occlusion (Figure 8). There was no distinguishable difference in interhemispheric rSO2 measurements between different reperfusion outcomes. The sensors did not obscure the view or negatively impact EVT. We experienced a significant number of sensor malfunctions, probably due to poor sensor adhesion.

**FIGURE 8:**
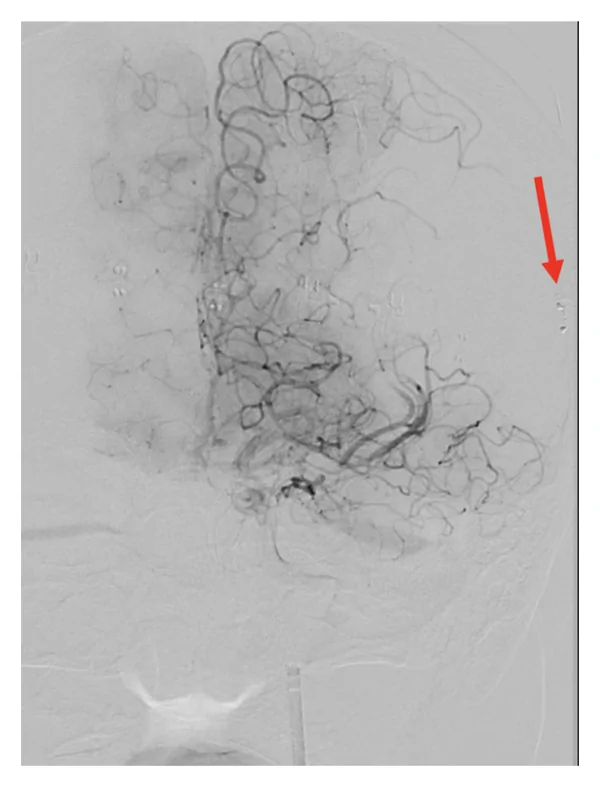
Digital subtraction angiography image of Patient 5 showing the NIRS sensor (red arrow) over the occluded MCA.

In patient #4, we observed a drop of approximately 10% points in rSO_2_ on the unaffected side, in both parietal and frontal sensors, lasting approximately 120 s starting just before the marked EVT time. Our results did not show significant interhemispheric differences that would help identify an LVO in other study patients. We hypothesise that the lack of change in rSO_2_ was due to the high proportion of venous blood on the brain’s surface. Hiramatsu et al. showed a clear pre‐ to post‐intervention difference in rSO_2_ in patients with ICA occlusion but not in those with distal MCA occlusion. They hypothesised that the difference was due to the small infarct size and that it might be possible to detect differences with parietally positioned sensors [15]. Our study could not show any difference, even with parietally placed sensors and M1 thrombi. Although recognising ICA occlusions would be reliable, it would not be sufficient for using NIRS prehospitally, as most LVO strokes are in the MCA.

Interestingly, four of five patients showed higher saturations on the affected side at the frontal sensors, although the difference was less than 10% points in all patients. This difference could be due to collateral flow through the anterior cerebral artery. Moreau et al. also found that cerebral saturations were higher in the frontal sensors on the affected side in stroke patients [23]. Despite the difference between frontal probes, there was no significant difference between frontal and parietal sensors on the affected side.

Moreau et al. showed that frequency‐domain NIRS (fdNIRS) may detect changes in cerebral oxygen saturation in stroke patients [23]. Giacalone et al. reported similar findings using time‐domain NIRS (tdNIRS) in stroke patients [24]. Our findings conform with those of Collette et al., who state that NIRS cannot reliably recognise LVO in the anterior circulation. This is due to two reasons: either there is no interhemispheric difference, or NIRS cannot detect it. The latter is more probable because patients in both studies had symptoms and positive imaging findings. Collette et al. hypothesised that the lack of difference might be due to the recording time being too short [25]. In our research, the NIRS recorded values between 1 and 4 h after the EVT, much longer than was chosen for the analysis, but no signal of any significant changes could be seen in the overall data. Also, the number of missing values increased. Finally, Collette et al. hypothesise that extracerebral tissues might affect rSO_2_ readings [25]. Another reason might be that the vast majority (approx. 70%) of the blood in the brain is on the venous side; because of this, the changes in arterial and capillary saturations cannot be seen.

Our primary goal was to test the feasibility of a cwNIRS device suitable for prehospital use in recognising LVO, rather than to evaluate the exact physiological mechanisms of cerebral ischaemia. Because this was a feasibility study to test a device for prehospital use, we intentionally wanted to keep our inclusion criteria wide, as the patient population prehospitally would be highly heterogeneous. Our findings suggest that cwNIRS lacks the reliability and accuracy required for prehospital LVO detection. Contradictory findings between studies on NIRS use for LVO detection and observation may reflect differences in NIRS technique (continuous‐wave, time‐domain and frequency‐domain), measurement protocols, anatomical targeting and specific study objectives.

Future research should explore alternative technologies, such as electroencephalography, volumetric impedance phase shift spectroscopy, cranial accelerometers and transcranial Doppler [26–28]. Transcranial Doppler has shown promise in detecting anterior circulation LVOs [28]. Other NIRS techniques, such as fdNIRS, show promise and could be of interest for future prehospital use as these devices become lighter and smaller.

### 4.1. Strengths and Limitations

To our knowledge, this was the first study employing an NIRS device suitable for prehospital use to look for a clinically significant difference in rSO_2_ in patients with LVO. This was also the first study to compare whether parietally positioned sensors are superior to frontally positioned sensors in detecting LVO.

The study included very few patients because the sensors were very expensive. The patients were highly heterogeneous in occlusion site, time to EVT and recovery measured by mRS. Half of the patients had partially missing data due to sensor‐related problems. Patient #3 was missing all measurements from the right frontal sensor, which we suspect was due to poor sensor adherence to the skin. Patient #5 missed only four values from the right parietal sensor post‐EVT. Patient #6 was missing 24 data points from the right parietal sensor pre‐EVT and all measurements post‐EVT. Patient #6 was also missing 20 measurements pre‐ and 70 post‐EVT from left parietal sensors. Missing data were probably due to poor sensor adherence to the skin or a sensor malfunction. Because of the missing data points, we could not compare the frontal measurements of patient #3 and the parietal measurements of patient #6. Across all patients, frontal sensor measurements failed in 150 of 1200 data points (13%), and parietal sensor measurements failed in 191 of 1200 data points (16%). Sensor placement was not correlated with sensor failure. Our measurement failure rate was comparable to studies by Moreau et al. and Giacalone et al., who used fdNIRS and tdNIRS [23, 24]. In the prehospital setting, clinicians would naturally try to fix obvious sensor issues, but in our study the need for time‐critical intervention was obvious, so we prioritised treatment over fixing the sensor problem.

We did not report extensively on physiological parameters such as MAP, oxygen saturation, FiO2, heart rate, ventilation status, etc. Changes in these parameters would undoubtedly affect perfusion and rSO2 in brain tissue, but global systemic influences would be expected to affect both hemispheres simultaneously and are therefore unlikely to explain the absence of interhemispheric differences between affected and unaffected hemispheres. Patient recruitment was terminated early because the importers could not provide more sensors.

## 5. Conclusions

In this pilot feasibility study, the evaluated NIRS configuration did not demonstrate a sufficiently consistent hemispheric signal for reliable identification of anterior circulation LVO during acute stroke treatment. While these findings do not rule out the potential utility of other optical monitoring approaches, they suggest that this specific implementation is unlikely to be suitable for prehospital LVO detection without substantial technical advances.

**Nomenclature**
CED: Conjugate eye deviation
CT: Computed tomography
CTA: Computed tomographic angiography
cwNIRS: Continuous‐wave near‐infrared spectroscopy
ED: Emergency department
EVT: Endovascular treatment
fdNIRS: Frequency‐domain near‐infrared spectroscopy
FIMEA: Finnish Medical Agency
ICA: Internal carotid artery
IQR: Interquartile ranges
LKW: Last known well
LVO: Large vessel occlusion
MAP: Mean arterial pressure
MCA: Middle cerebral artery
mRS: Modified Rankin Scale
MT: Mechanical thrombectomy
NIRS: Near‐infrared spectroscopy
rSO_2_
: Regional oxygen saturation
tdNIRS: Time‐domain near‐infrared spectroscopy

## Author Contributions

All authors took part in planning the study. Pauli Vuorinen, Jyrki Ollikainen and Pasi Jolma gathered the data. Joonas Kiili, the corresponding author, was responsible for the data analysis and writing the original draft of the manuscript. All authors contributed equally to the review and editing of the draft.

## Funding

This study was partially supported by the State funding for university‐level health research, Tampere University Hospital, Wellbeing Services County of Pirkanmaa Project number 9AB009. Joonas Kiili received a personal grant from Maire Taponen’s foundation.

## Ethics Statement

This study was approved by the Ethics Committee of Tampere University Hospital (ETL R21159L), and access to patient registries was granted by the Medical Directors of the departments involved.

The CE‐approved Nonin SenSmart Model X‐100 (NONIN Medical Inc. Plymouth, Minnesota, USA) NIRS device was used for this study. As a device study, this study required permission from the Finnish Medical Agency (FIMEA). Any adverse effects (MDR Article 2(57)), serious adverse effects (MDR Article 2(58)) and protocol deviations, which we had none in this study, would have been documented on a separate research form and reported to the ethical board and FIMEA (MDCG 2020‐10/1 Safety reporting in clinical investigations of medical devices under the Regulation (EU) 2017/745 May 2020). The study protocol required attaching sensors to the patient’s head (frontally and parietally), which may have required removing hair in the sensor area, similar to an electrocardiogram. The inconvenience for the patient was only cosmetic and short term, as the hair would grow back. This study was carried out in accordance with the World Medical Association Declaration of Helsinki of 1964, revised at the 64th General Assembly in Brazil in 2013.

## Consent

Delayed consent was collected from the study patients or their legal representatives as soon as possible. This study was considered emergency research because eligible patients could not provide consent before treatment due to their serious condition.

The article contains no individual information.

## Conflicts of Interest

The authors declare no conflicts of interest.

## Data Availability

The datasets used and/or analysed during the current study are available from the corresponding author upon reasonable request.
